# Disposable facemask waste combustion emits neuroactive smoke particulate matter

**DOI:** 10.1038/s41598-023-44972-0

**Published:** 2023-10-18

**Authors:** Artem Pastukhov, Konstantin Paliienko, Natalia Pozdnyakova, Natalia Krisanova, Marina Dudarenko, Lilia Kalynovska, Alla Tarasenko, Olena Gnatyuk, Galina Dovbeshko, Tatiana Borisova

**Affiliations:** 1 Palladin Institute of Biochemistry, National Academy of Sciences of Ukraine, 9 Leontovicha Str, Kyiv, 01054 Ukraine; 2 Institute of Physics, National Academy of Sciences of Ukraine, Prospect Nauky 46, Kyiv, 03028 Ukraine

**Keywords:** Neurochemistry, Fire ecology, Environmental impact, Nanoparticles

## Abstract

Tremendous deposits of disposable medical facemask waste after the COVID-19 pandemic require improvement of waste management practice according to WHO report 2022, moreover facemasks are still in use around the world to protect against numerous airborne infections. Here, water-suspended smoke preparations from the combustion of disposable medical facemasks (polypropylene fibers) were collected; size, zeta potential, surface groups of smoke particulate matter were determined by dynamic light scattering, FTIR and Raman spectroscopy, and their optical properties were characterized. Neurochemical study using nerve terminals isolated from rat cortex revealed a significant decrease in the initial rate of the uptake/accumulation of excitatory and inhibitory neurotransmitters, L-[^14^C]glutamate and [^3^H]GABA, and exocytotic release, and also an increase in the extracellular level of these neurotransmitters. Fluorescent measurements revealed that ROS generation induced by hydrogen peroxide and glutamate receptor agonist kainate decreased in nerve terminals. A decrease in the membrane potential of nerve terminals and isolated neurons, the mitochondrial potential and synaptic vesicle acidification was also shown. Therefore, accidental or intentional utilization of disposable medical facemask waste by combustion results in the release of neuroactive ultrafine particulate matter to the environment, thereby contributing to plastic-associated pollution of air and water resources and neuropathology development and expansion.

## Introduction

The burden of neurological disorders, one of the main causes of disability and premature death in Europe, is expected to increase, and moreover it can be significantly aggravated by COVID pandemic because of “long COVID” neurological complications^1–3^. Stable expansion of neurological disorders and complications, aetiology of which still remains unclear, is linked to air pollution by PM_2.5_ (Particulate Matter with the size less than 2.5 μm) that is capable of target the nervous system, thereby triggering development of lowered cognitive function, autism, neurodegenerative disease, dementia, stroke, etc.^4^. This is a global concern because PM is dispersed around the world, traveling across oceans and continents^5,6^.

Plastic waste utilization was considered a main environmental problem due to growing global concerns about worldwide pollution even before the COVID-19 pandemic^7^. Since the primary way of coronavirus transmission according to WHO is the droplet route, most countries sanctioned regulations for obligatory use of personal disposable protective medical facemasks in order to prevent the spread of the infection and to protect against COVID-19. Because of this, the demand for these facemasks significantly increased in 2020. After the COVID-19 pandemic, the facemasks are still used by the population around the world in daily life in order to protect against numerous airborne infections. Disposability of the facemasks reduces the contamination risks as compared to the reusable ones^8–10^.

According to WHO report 2022, thousands of tonnes of extra medical waste from the COVID-19 pandemic has put tremendous strain on waste management systems worldwide, threatening environment and human health and underlying critical needs to improve waste management practices^11^. Huge amount of used facemasks requires establishment of disposal routes to reduce the harmful effects of their waste on the environment^7^. 30% of healthcare facilities in developed countries and 60% in the least developed ones are not equipped to handle existing waste loads and moreover the additional COVID-19 load. Current literature shows that the huge number of contaminated facemasks is a problem for the environment, as the current processes of disposal (i.e. incineration and reclamation) are accompanied by the release of toxic chemicals^12,13^. Poorly managed landfills and waste disposal sites potentially impact communities through contaminated air from burning waste, and poor water quality^11^. Accidental burning of used facemasks can also occur during fires of the domestic mixed facemask-containing garbage, and besides that intentional uncontrolled illegal utilisation of the facemask waste by combustion is still of practice in the least developed countries.

There are plenty of biopolymers available in the market, whereas most disposable single-use facemasks are made from fossil-derived polymers^14,15^. The standard textile used to mass-produce the disposable medical facemasks is polypropylene sheets that are made using spun-bond or melt-blown technology^16^. Material recycling of used facemask waste is impossible, as they can be contaminated with pathogens and have to be considered and handled as hazardous waste, and so thermal utilization offers a reliable disposal route for used facemasks^8–10^.

Under laboratory conditions, we have recently synthesized the water-**s**uspended **s**moke samples from plastics that are appropriate for neurochemical testing using rat brain nerve terminals. These preparations contained major nano-sized (30 nm) PM fraction and impaired functioning of neurotransmitter transporters, thereby provoking presynaptic malfunctioning and triggering neurological consequences^17^. It should be underlined that impairment of transport of excitatory and inhibitory neurotransmitters in the central nervous system, glutamate and γ-aminobutyric acid (GABA), respectively, is involved in the pathogenesis of major neurological complications and disorders. Also, a comparative neurochemical study of wood sawdust and plastic smoke particulate matter preparations revealed higher riskiness in mitigation of synaptic inhibition by plastic preparations as compared to the wood sawdust ones^18^.

Taking into account a high significance and globalism of disposable facemask waste utilization problem, the aims of this study were: (1) to collect in the laboratory conditions water-suspended smoke PM preparations of disposable medical facemasks from polypropylene fibers (mask preparations (MPs)) using previously developed methodical approach^17^; (2) to define the average size and Z-potential of particles in MPs using dynamic light scattering; (3) to analyse optical, fluorescence and surface properties of particles in MPs using Raman and Fourier-transform infrared (FTIR) spectra; (4) to assess neurotoxic properties of MPs studying key characteristics of synaptic neurotransmission in isolated rat cortex nerve terminals (synaptosomes), namely Na^+^-dependent transporter-mediated uptake and release, exocytosis and the ambient levels of L-[^14^C]glutamate and [^3^H]GABA; (5) to analyze MP effects on the membrane potential of synaptosomes and isolated cortex neurons, mitochondrial potential, synaptic vesicle acidification and stimulated generation of reactive oxygen species (ROS) using fluorescent dyes rhodamine 6G, mitochondrial membrane potential assay kit JC-1, acridine orange and 2′,7′-dichlorofluorescein (DCF), respectively.

## Results

### Size of particles in MPs and their Z-potential, surface, optical and fluorescent properties

#### Dynamic light scatter

**Figure 1:**
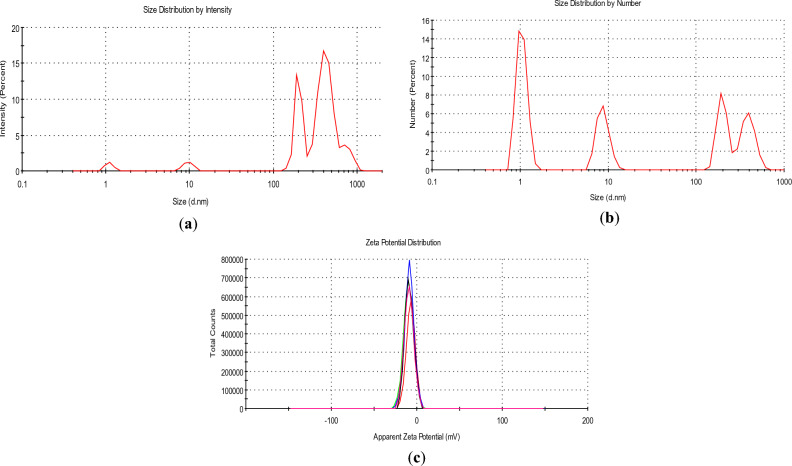
Dynamic light scattering diagrams of MPs. (**a**) Size distribution of MPs by intensity. (**b**) Size distribution of MPs by number. The graphs represent cumulative data analysis of five consequent measurements, each for 2 min. (**c**) Graph of Z-potential distribution between 5 measurements, pH 3.75, and each measurement included 50 runs for 10 s. Red line—first measurement, green—second, blue, black and pink—next following, correspondingly.

MPs were a colloid obtained by filtering through a glass microfiber filter with a pore diameter of 1.0 µm to eliminate debris and enrich preparation with nanoscale fractions. MP colloid was a heterogeneous system with a high index of polydispersity (mean value of 5 measurements was 0.8); the samples contained particles of different hydrodynamic sizes. Figure 1a shows particle size distribution by intensity as a direct value. Figure 1b represents particle distribution by number, and several populations of nanoparticles of different size were observed, in particular MPs contained four fractions of a wide nano-sized range with mean values of hydrodynamic diameter equaled to 396 nm, 190 nm, 10 nm and 1.2 nm. Distribution by number showed a relatively equal quantity of particles in each population, with slightly increased content of ultrasmall particles. It differed from particle size distribution by intensity due to the impact of large particles in signal formation on the detector.

Z-potential, i.e. net charge of a nanoparticles’ surrounding in solvent, was assessed in the next series of the experiments that can indicate a stability of nanoparticles in the colloid. Z-potential of MPs under stock acidic pH 3.75 was equal to − 9.2 ± 5.4 mV (Fig. 1c). So, particles in this colloid had very low net charge that in turn could lead to their coagulation and aggregation in acidic media. Z-potential of MPs was measured also in basic medium at pH 10.1 and had a value of − 24.0 ± 4.9 mV. It means that the system contained carbon nanoparticles with negatively-charged chemical groups at the surface and become more stable at higher pH values.

#### FTIR and Raman spectroscopy

**Figure 2:**
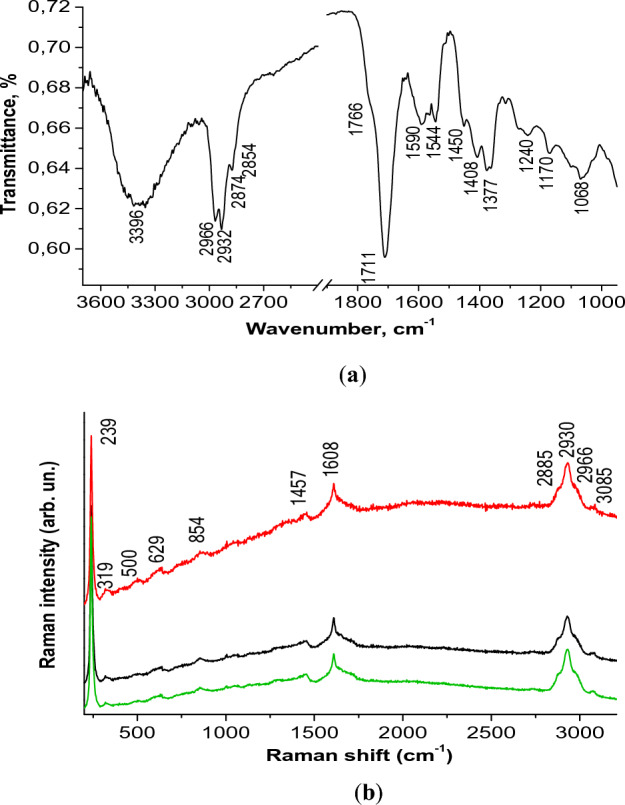
FTIR (**a**) and Raman spectra (**b**) of MPs.

The spectrum of MPs indicated the presence of two products, i.e. short-chain alcohols (R–C–OH) and carboxylic acids (R-COOH)^19^. Analysis of the position of the infrared (IR) absorption bands (Fig. 2a) from left to right revealed the band in the region 3396 cm^−1^, which referred to the stretching vibrations of OH^20^, and a set of peaks at 2966, 2932, 2872, 2854 cm^−1^, related to CH stretching vibrations of hydrocarbons^20,21^. A characteristic feature was that the CH bands were not in the form of a shoulder along with the OH chain, but looked like practically separated bands. It was difficult to estimate the length of the hydrocarbon chain according to IR spectra, however the ratio of intensity of CH_3_ at 2966 cm^−1^ to CH_2_ at 2932 cm^−1^ indicated a greater contribution of CH_2_. It can be assumed that the C–C chain was short, since both CH_3_ bands symmetric and asymmetric were pronounced, and the symmetrical CH_2_ at 2854 cm^−1^ was only in the form of a small shoulder^21^. The position of the band C=O at 1711 cm^−1^ and the broad OH band at 3396 cm^−1^ were markers of the presence of a carboxyl molecular group, which was a part of saturated carboxylic acids^22,23^. The shoulder at 1766 cm^−1^ indicated the presence of monomers with hydrogen bonds, and carbonic acid dimers with hydrogen bonds (band at 1711 cm^−1^)^24,25^. The band at 1590 cm^−1^, assigned to the molecular group C=C, can be attributed to carbon particles formed during the combustion of organic compounds.

Raman spectra complement the information received from FTIR spectra, since different types of vibrations were active in FTIR and Raman (Table 1). In Raman spectroscopy, the spectrum of MPs registered characteristic markers of CH stretching vibrations in the region at 2966, 2930, 2885 cm^−1^, as well as a band at 3085 cm^−1^, which refers to C–H in C=C–H group in compounds with rings, and the band of CH_2_ deformation vibrations near 1457 cm^−1^ (Fig. 2b).

**Table 1:** Assignment of the main spectrum bands to molecular groups according to^19–26^ (def., deformation vibrations; str, stretching vibration; sh, shoulder; w, weak; m, medium; s, strong; vs, very strong; vvs, super strong; sym, symmetrical; asym, asymmetrical vibrations).

Raman spectrum		IR spectrum	
Position, cm^−1^	Assignment	Position, cm^−1^	Assignment
319 (321^26^)	CaF_2_ substrate	1068	C–C-O str, C-CH_3_
500 (530^26^)		1170 (1167 sh^26^)	C–C str, def CH
629	no in propylene	1240, no in propylene	C-O str, OH def
854 (841 vs^26^)		1377 (1370 s)	CH_3_ def^21^, CH
		1408 no in propylene	C-O–H def, CH_2_
1457 (1458 vs^26^)	CH_2_ def, CH_3_ asym def	1450 (1460s^26^)	CH_2_ def, CH_3_ asym def
		1544, no in propylene	C=C str
1608	C–C str	1590, no in propylene	C=C str, COO- str
		1711, no in propylene	C=O str dimers
		1766, no in propylene	C=O str monomers
		2854 (2840 vs^26^)	CH_2_ str sym^21^
2885 (2883 s^26^)	CH srt	2874 (2887 vs^26^)	CH_3_ str sym^21^
2930 (2920 m^26^)	CH str	2932 (2921 vvs^26^)	CH_2_ str asym^21^
2966	CH str	2966 (2956 vvs^26^)	CH_3_ str asym^21^
3085	CH ring		
		3396, no in propylene	OH str

#### Optical and fluorescent properties

**Figure 3:**
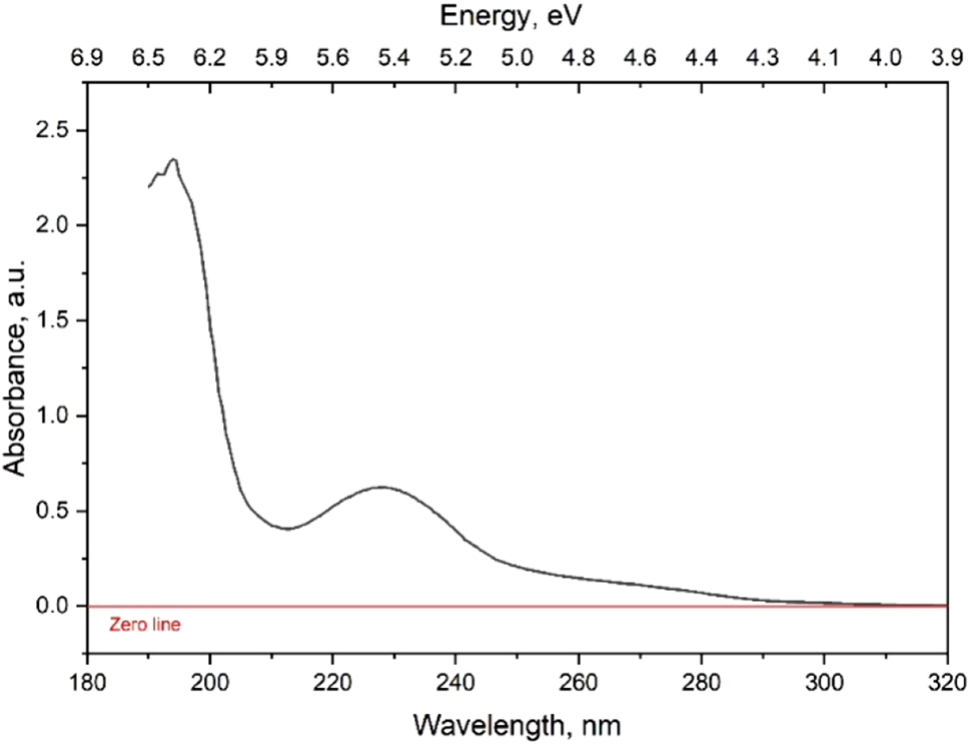
UV–VIS absorption spectrum of MPs in water.

The optical properties of MPs were characterized by ultraviolet–visible (UV–Vis) absorption and photoluminescent spectroscopy. Absorption of MPs was mainly registered in the UV region (range of 190–300 nm) (Fig. 3). The main light absorption has occurred at 194 nm and 228 nm. These wavelengths correspond to absorption of light with high energies (6.4 and 5.45 eV, respectively). Such shift of absorption into high-energy range could be explained by the presence of organic matter with π–π* transitions inside, involving unsaturated *sp*^2^ carbons (e.g., C=C bonds)^27^. There were no or very low number of molecules capable of absorbing lower energy wavelengths. Despite relatively intense absorption, the samples were found to be non-fluorescent. Such properties tended to possess small non-aromatic organic molecules with multiple conjugated double bonds, often containing carbonyl or nitrogen groups in their structure, which contribute to their strong UV absorption (e.g., ketones, aldehydes, polyenes, etc.)^28,29^.

### Neuroactive properties of MPs examined using animals and radiolabelled neurotransmitters

#### The transporter-mediated uptake of L-[^14^C]glutamate and [^3^H]GABA by the nerve terminals in the presence of MPs

**Figure 4:**
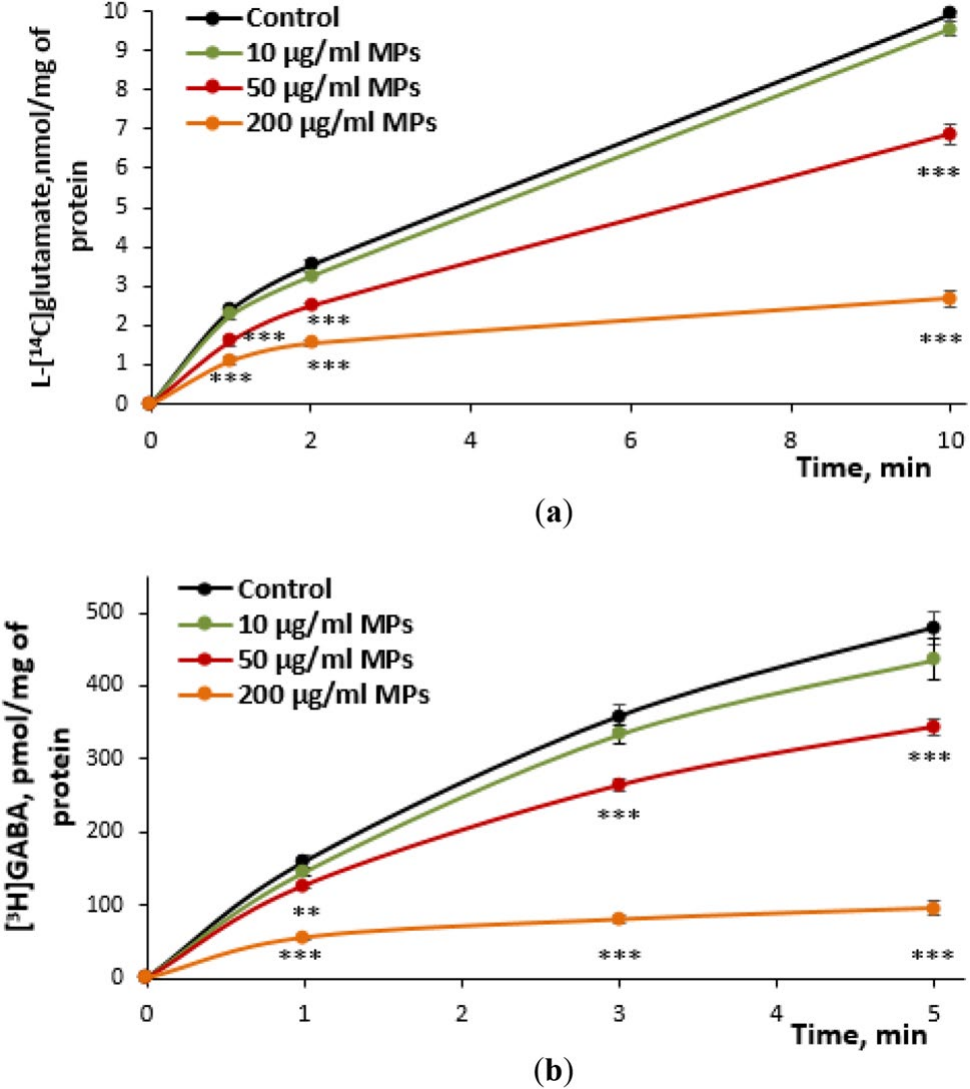
The time course of the transporter-mediated uptake of L-[^14^C]glutamate (**a**) and [^3^H]GABA (**b**) by the nerve terminals in the presence of MPs at concentrations of 10, 50 and 200 μg/ml. Data are presented as mean ± SEM. **p < 0.01; ***p < 0.001 as compared to the control; n = 15.

In the first series of neurotoxicological studies, the effects of MPs on the initial rate of the transporter-mediated uptake and accumulation of excitatory and inhibitory neurotransmitters by the nerve terminals were assessed. The transporter-mediated uptake is one of key processes in the presynapse that determines the proper extracellular level of neurotransmitters in the synaptic cleft^30,31^. It was shown that both parameters, i.e. the initial rate of the uptake and accumulation of L-[^14^C]glutamate for 10 min, were not altered by MPs at a concentration of 10 μg/ml and significantly reduced in a dose-dependent manner at 50 μg/ml and 200 μg/ml MPs (Fig. 4a). The initial rate of the uptake and accumulation of [^3^H]GABA by nerve terminals were also insignificantly reduced (a tendency to reduce) by MPs at a concentration of 10 μg/ml, whereas these parameters considerably decreased in a dose-dependent manner at 50 μg/ml and 200 μg/ml MPs (Fig. 4b).

#### The extracellular level of L-[^14^C]glutamate and [^3^H]GABA in the nerve terminal preparations in the presence of MPs

The extracellular level of neurotransmitters between the episodes of exocytotic release is a dynamic balance of transporter-mediated uptake/release and the tonic leakage of neurotransmitters. This is a very important synaptic parameter that determines strength of synaptic contacts and tonic neuronal communication; regulates synaptic neurotransmission and is tightly associated with the energetic status and the membrane integrity of nerve terminals^30,31^. As shown in Table 2, the extracellular level of L-[^14^C]glutamate and [^3^H]GABA in the nerve terminal preparations was not changed in the presence of MPs at concentrations of 10 μg/ml and 50 μg/ml, whereas significantly increased at 200 μg/ml. These data are in accordance with above L-[^14^C]glutamate and [^3^H]GABA uptake results. In particular, the lesser the uptake was, the higher the extracellular level can be registered.

**Table 2:** The extracellular level of L-[^14^C]glutamate and [^3^H]GABA in the nerve terminal preparations in the presence of MPs at concentrations of 10, 50 and 200 μg/ml.

	The extracellular level of L-[^14^C]glutamate in the nerve terminal preparations (% of total accumulated label)	F;* p* value n = 15	The extracellular level of [^3^H]GABA in the nerve terminal preparations (% of total accumulated label)	F;* p* value n = 15
Control	19.03 ± 0.99		15.77 ± 0.39	
10 µg/ml MPs	18.87 ± 0.86	F_(1,28)_ = 0.01; * p* = 0.90; n.s	15.12 ± 0.56	F_(1,28)_ = 0.95; * p* = 0.34; n.s
50 µg/ml MPs	18.30 ± 0.72	F_(1,28)_ = 0.37; * p* = 0.54; n.s	16.35 ± 0.33	F_(1,28)_ = 1.39; * p* = 0.24; n.s
200 µg/ml MPs	39.48 ± 0.80***	F_(1,28)_ = 276.68; * p* < 0.001;	30.54 ± 0.74***	F_(1,28)_ = 341.57; * p* < 0.001

***p < 0.001; n.s., no significant differences as compared to the appropriate control.

#### Tonic release of [^3^H]GABA from the nerve terminals in the presence of MPs

Tonic release of [^3^H]GABA from the nerve terminals measured during blockage of GABA transporters by selective inhibitor NO-711 reflected the membrane permeability to small molecules. As shown in Table 3, tonic release of [^3^H]GABA from the nerve terminals in the presence of MPs at concentrations of 10 and 50 μg/ml was not changed significantly, whereas an increase in the MP concentration up to 200 μg/ml resulted in significant growth of this parameter.

**Table 3:** Tonic release of [^3^H]GABA from the nerve terminals in the presence of MPs at concentrations of 10, 50 and 200 μg/ml.

	Tonic release of [^3^H]GABA from the nerve terminals (% of total accumulated label)	F;* p* value n = 15
Control	21.67 ± 0.66	
10 µg/ml MPs	21.90 ± 0.84	F_(1,28)_ = 0.05; * p* = 0.82; n.s
50 µg/ml MPs	21.29 ± 0.81	F_(1,28)_ = 0.14; * p* = 0.71; n.s
200 µg/ml MPs	27.65 ± 0.72***	F_(1,28)_ = 40.1; * p* < 0.001

***p < 0.001; n.s., no significant differences as compared to the control.

#### Exocytotic release of neurotransmitters from the nerve terminals in the presence of MPs

**Figure 5:**
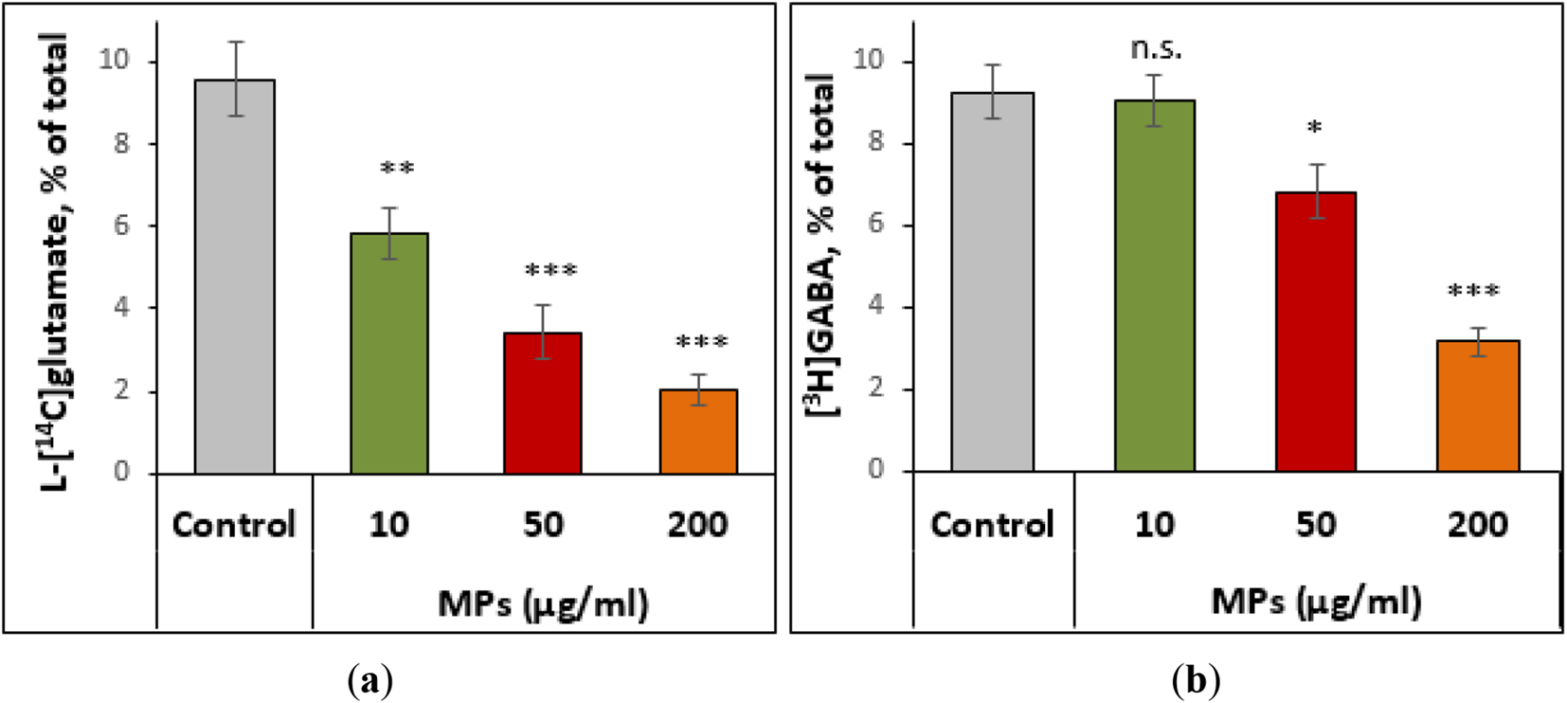
Exocytotic release of L-[^14^C]glutamate (**a**) and [^3^H]GABA (**b**) from the nerve terminals in the presence of MPs at concentrations of 10, 50 and 200 μg/ml. Data are presented as mean ± SEM. *p < 0.05; **p < 0.01; ***p < 0.001; n.s., no significant differences as compared to the control, n = 15.

Stimulated compound exocytosis, the last step of which includes a fusion of synaptic vesicles with the plasma membrane and release of their context to the synaptic clefts, is the main mechanism of synaptic neurotransmission. As shown in Fig. 5a, exocytotic release of L-[^14^C]glutamate from the nerve terminals significantly decreased in a dose-dependent manner in the presence of MPs at concentrations of 10 and 50 μg/ml. An increase in the MP concentration up to 200 μg/ml caused almost complete leakage of L-[^14^C]glutamate from the nerve terminals, and so negligible exocytotic release. Exocytotic release of [^3^H]GABA from the nerve terminals was not changed in the presence of 10 μg/ml MPs, and decreased in a dose-dependent manner in the presence of MPs at concentrations of 50 and 200 μg/ml (Fig. 5b).

### Fluorescence experiments on the assessment of the membrane potential of the nerve terminals and neurons, mitochondrial membrane potential and ROS generation in the presence of MPs

#### The membrane potential of the nerve terminals and isolated cortex neurons in the presence of MPs

**Figure 6:**
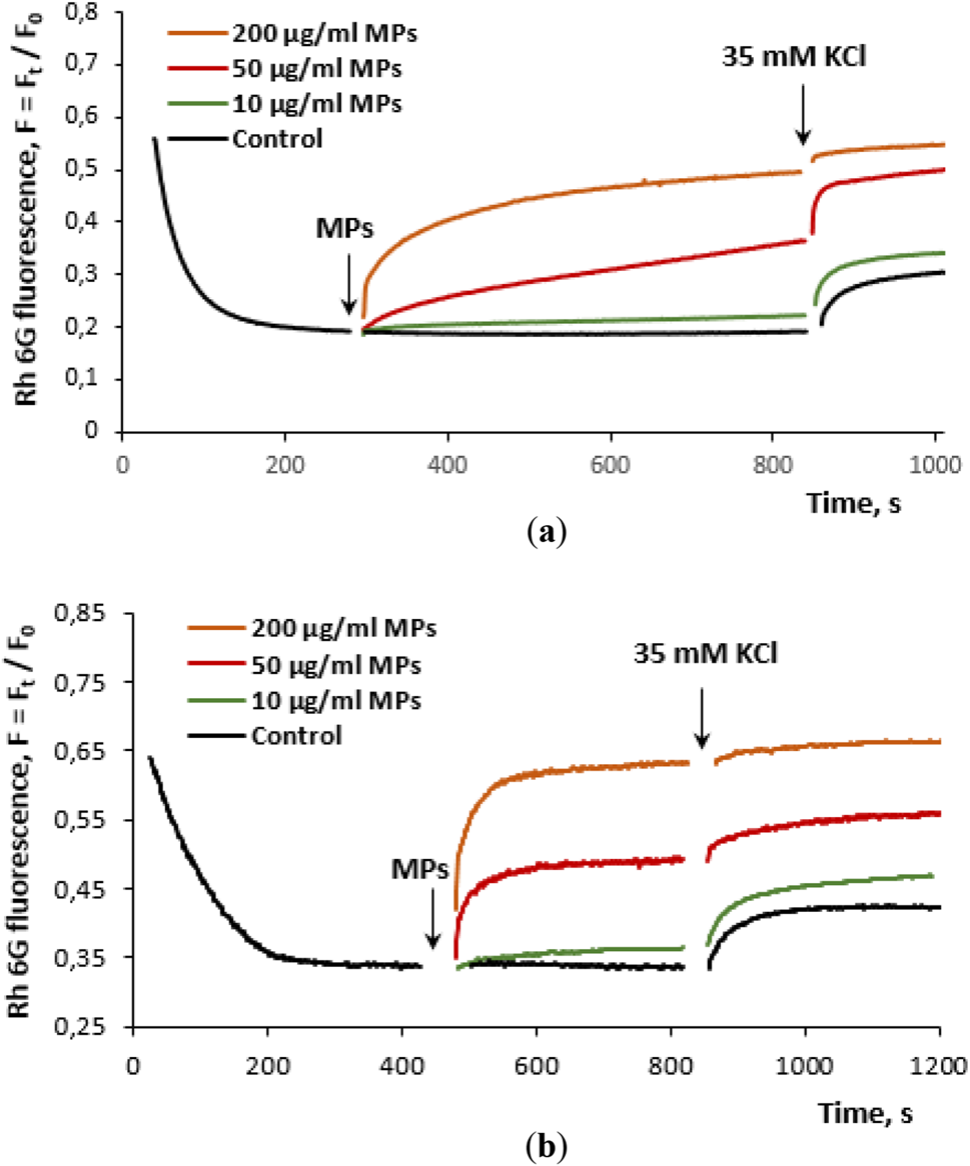
Fluorescence experiments**:** (**a**) The effects of MPs at concentrations of 10, 50 and 200 μg/ml on the membrane potential of the nerve terminals. (**b**) The effects of MPs at concentrations of 10, 50 and 200 μg/ml on the membrane potential of isolated cortex neurons. Synaptosome or neuron suspensions were equilibrated with rhodamine 6G (0.5 μM); when the steady level of the fluorescence was reached, MPs (marked by arrow) were added to synaptosome or neuron suspension. The trace represents 15 experiments performed with different preparations.

The potential of the plasma membrane of nerve cells is mainly responsible for proper transporter-mediated uptake, the extracellular level and exocytotic release of the neurotransmitters. It corresponds to the membrane integrity and reflects whether or not tested compounds cause membrane depolarization. The membrane potential of the nerve terminals and neurons isolated from the cortex was monitored using a potential-sensitive fluorescent dye rhodamine 6G. After the addition of the dye to the suspension of synaptosomes or neurons, quenching of the dye fluorescence was observed due to partial binding to a negative charge in the inner near membrane space of synaptosomes or neurons. It was shown that MPs at a concentration of 10 μg/ml did not influence both the synaptosomal and neuronal membrane potentials, whereas an increase in the MP concentration up to 50 and 200 μg/ml depolarized the synaptosomal and neuronal membranes in a dose-dependent manner (Fig. 6a,b).

#### The mitochondrial membrane potential of the nerve terminals in the presence of MPs

**Figure 7:**
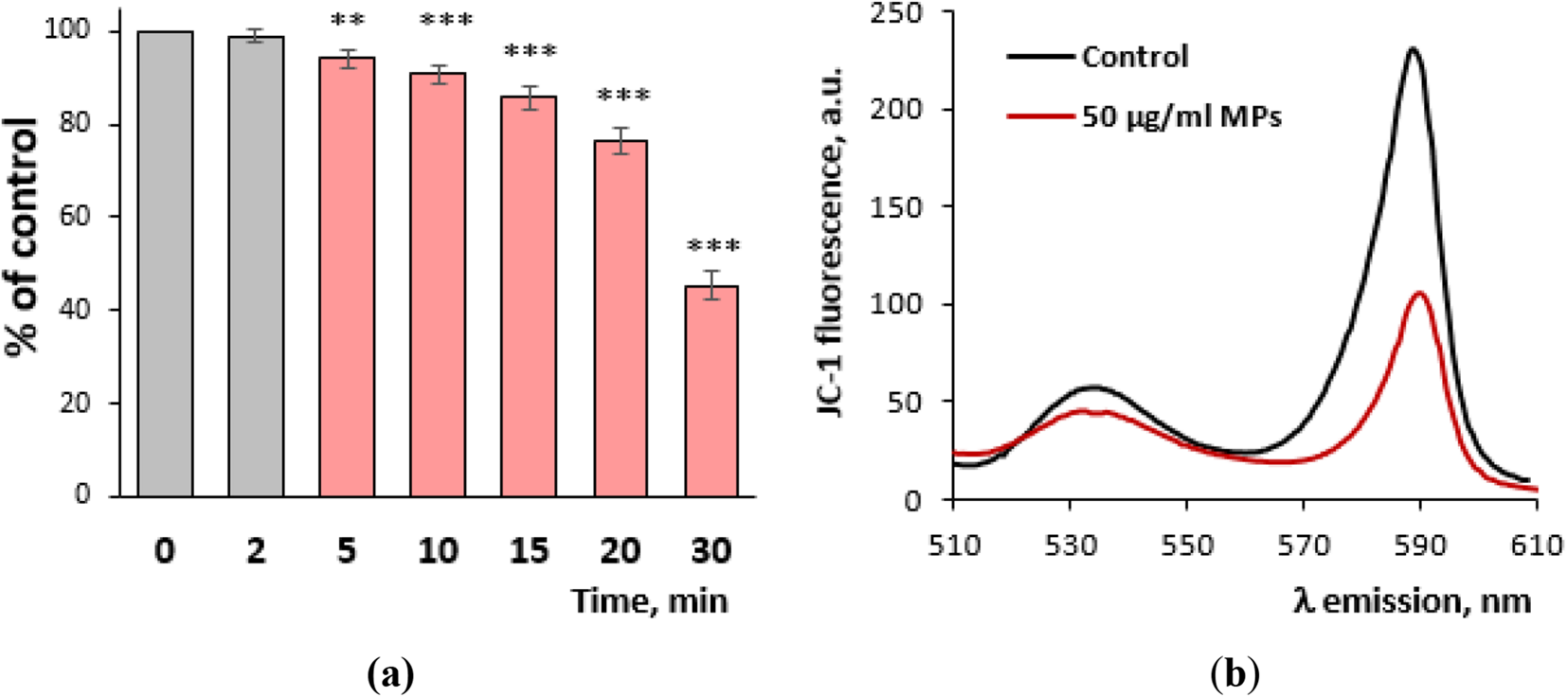
The mitochondrial membrane potential of the nerve terminals in the presence of MPs measured using the cationic membrane-permeable dye JC-1. (**a**) The time course of changes in JC-1 fluorescence. The dye (a final concentration of 2 µM) was added to the synaptosome suspension (a final protein concentration of 0.15 mg/ml), incubated for 10 min in the dark at a temperature of 37 °C. Then, MP aliquots were added to the cuvette and the kinetics of probe fluorescence was monitored for 30 min at an excitation wavelength of 485 nm and an emission wavelength of 590 nm. (**b**) JC-1 fluorescence spectra in the control and in the presence of MPs at a concentration of 50 μg/ml at 30 min time point recorded at an excitation wavelength of 485 nm and an emission wavelength from 510 to 610 nm. The trace represents 15 experiments performed with different preparations. (**a**) **p < 0.01; ***p < 0.001; as compared to the 0 min; n = 15.

In the next series of the experiments, the mitochondrial membrane potential was analyzed in the nerve terminals using a cationic carbocyanine fluorescent dye JC-1 accumulated by mitochondria. Before kinetic measurements and the addition of MPs, JC-1 fluorescence spectra of each synaptosome sample were recorded at 0 min time point. The fluorescence spectra were similar at 0 min time point in all samples. For quantitative analysis, the ratio of the fluorescence intensity at 590 and 530 nm was used. This ratio makes it possible to overcome the following problems, such as the difference in the probe loading, the number of mitochondria, and MP-associated fluorescence quenching. JC-1 fluorescence spectra in the control and in the presence of MPs at a concentration of 50 μg/ml were monitored at a 30 min time point. In the control, the probe spectrum was revealed to be unchanged for 30 min. As shown in Fig. 7a,b, MPs at a concentration of 50 μg/ml caused depolarization of the mitochondria membrane in the nerve terminals. The ratio of the fluorescence intensity at 590 versus 530 nm in the control was 3.99, whereas it has become 1.82 in the presence of MPs that reflected more than a twofold decrease in the mitochondrial membrane potential.

#### The acidification of synaptic vesicles in the presence of MPs

**Figure 8:**
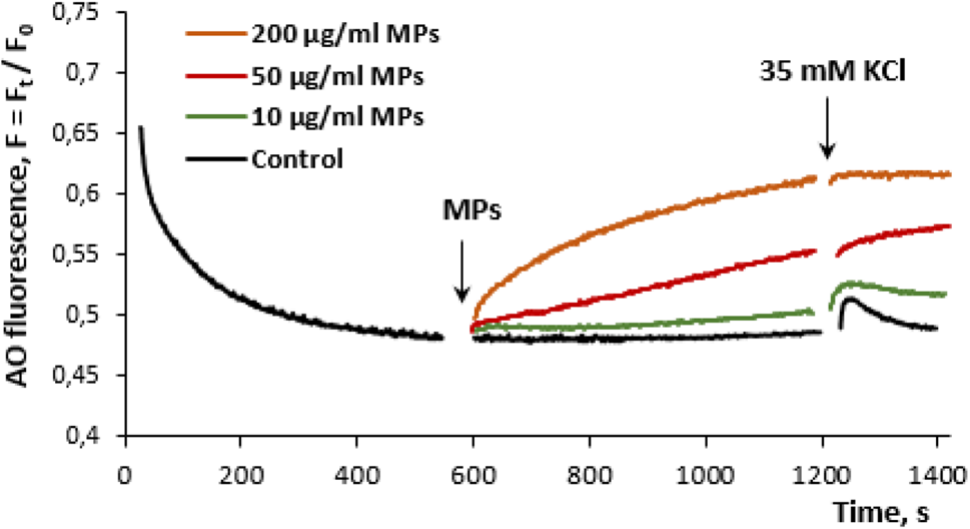
Synaptic vesicle acidification in the presence of MPs at concentrations of 10, 50, 200 μg/ml. The synaptosome suspension was equilibrated with 5 µM acridine orange, and when the steady level of the dye fluorescence had been reached, MP aliquots (marked by arrow) were applied. The trace represents 15 experiments performed with different preparations.

Taking into account the harmful influence of MPs on the exocytosis shown in Fig. 5, the effect of MPs on synaptic vesicle acidification was analysed using the pH sensitive fluorescence probe acridine orange (Fig. 8). The probe was accumulated by synaptic vesicles inside of the nerve terminals in concordance with their pH gradient. Quenching of the fluorescence during incubation with the nerve terminals reflected the accumulation of the probe by synaptic vesicles. An addition of acridine orange (5 μM) to the synaptosomal suspension resulted in setting of a steady state level of the fluorescence in 10 min. Application of MP aliquots dissipated the proton gradient of synaptic vesicles in the nerve terminals in a dose-dependent manner starting from the concentration of 10 μg/ml (Fig. 8). An increase in the fluorescence showed the release of the probe to the extracellular space during vesicle exocytosis or dissipation of the pH gradient because of different reasons. As shown in Fig. 8, 35 mM KCl-induced membrane depolarization resulted in a spike of the fluorescence due to a temporal decrease in nerve terminal acidification associated with exocytosis. An increase in the MP concentration was associated with a decrease in the fluorescence spike that reflected reduced exocytosis, thereby confirming above experiments with radiolabeled neurotransmitters presented in Fig. 5.

#### ROS generation in the nerve terminals in the presence of MPs

**Figure 9:**
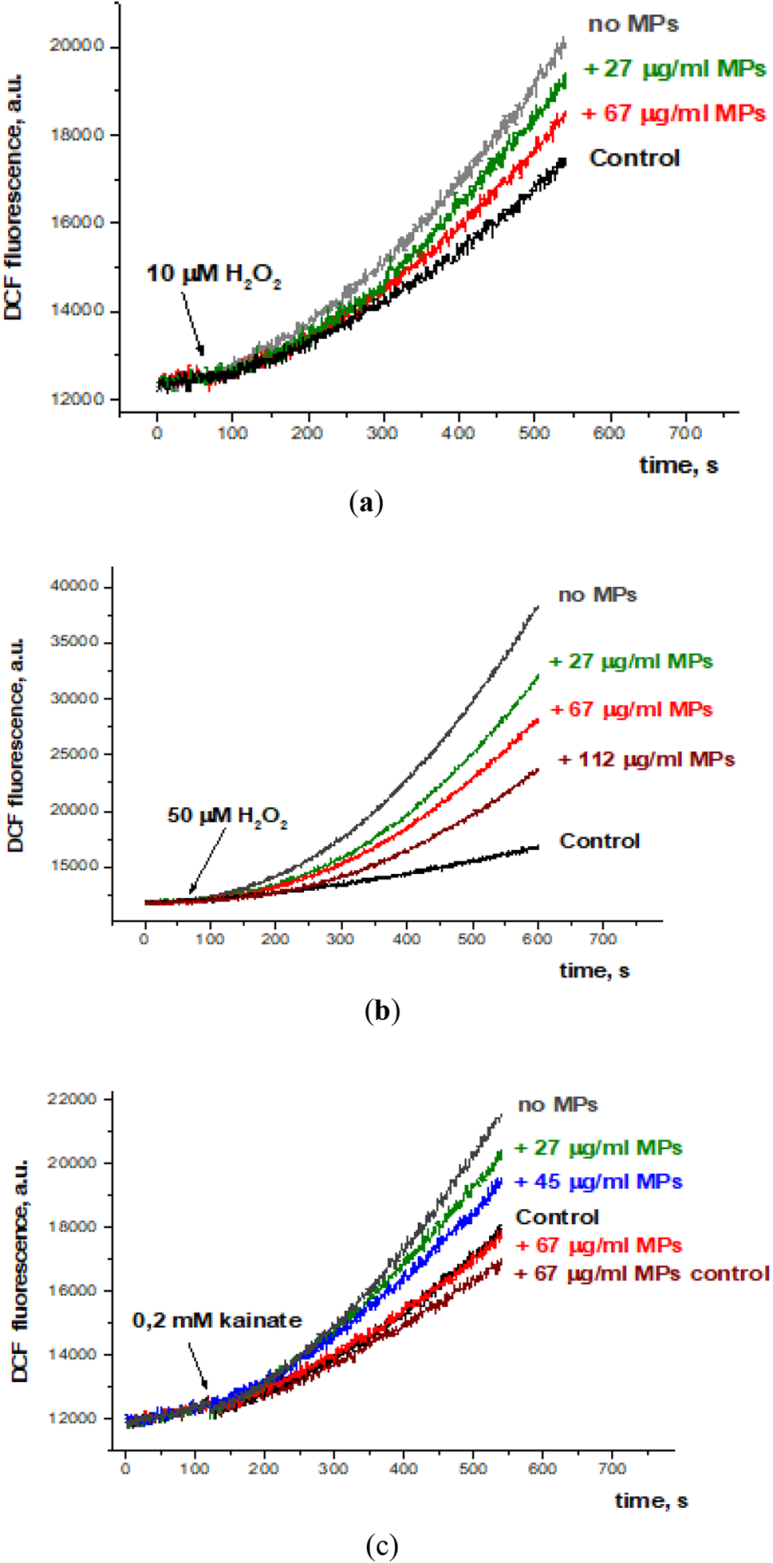
Effects of MPs on ROS generation in the nerve terminals induced by H_2_O_2_ at concentrations of 10 μg/ml (**a**) and 50 μg/ml (**b**) and by kainate at a concentration of 0.2 mM (**c**). Synaptosomes were pre-incubated with MPs at concentrations of 27, 45, 67, and 112 μg/ml, and then H_2_O_2_ (10 or 50 μM) or kainate (0.2 mM) were added. Each trace is representative of 15 experimental data records performed with different synaptosome preparations.

Kinetics of H_2_O_2_—and kainate (agonist of kainate/AMPA type glutamate receptors)—induced ROS generation in the nerve terminals was monitored using fluorescent dye 2′,7′-dichlorodihydrofluorescein diacetate (H_2_-DCFDA). Figure 9a,b represents a dose-dependent inhibiting effect of MPs at different concentrations on ROS generation stimulated by 10 and 50 μM H_2_O_2_. Figure 9c demonstrates the dose-dependent inhibiting effect of MPs at different concentrations on the level of intrasynaptosomal ROS, which was generated in response to the addition of kainate (0.2 mM). Therefore, MPs were able to reduce not only exogenous ROS added to synaptosomes (like H_2_O_2_), but also ROS generated due to activation of intracellular signaling pathways (like kainate).

## Discussion

With this study, we are contributing towards the urgent and global problem of utilization of disposable medical facemask deposits underlined in WHO report 2022^11^. To overcome the problem of disposable facemask utilization, it is necessary to determine their degradation and flammability characteristics for efficient waste management^12^. Here, data on dynamic light scattering revealed that MPs were heterogeneous and consisted of four populations of nanoparticles. Distribution by number showed high content of ultrasmall particles with negatively-charged chemical groups at the surface (Fig. 1). According to the literature data, ultrafine air pollution PM_0.1_ (less than 0.1 µm in diameter) is more harmful to human health as compared to fine PM_2.5_ and coarse PM_10_ (less than 10 µm in diameter) ones. The lesser the diameter of air pollution PM is, the higher toxicity via mechanisms of oxidative stress and inflammation can be registered^32^.

In this study, it was demonstrated that MPs decreased the transporter-mediated uptake of L-[^14^C]glutamate and [^3^H]GABA, and exocytotic release, as well as increased the extracellular level of both neurotransmitters. MP-induced changes in these characteristics were in accordance with each other and represented experimental “cross-check” parameters for assessment of synaptic neurotransmission. For example, a MP-induced increase in the extracellular level of neurotransmitters (a balance of uptake and tonic release) (Table 2) agreed with decreased synaptosomal neurotransmitter uptake by MPs (Fig. 4). It should be noted that an increase in the extracellular level of glutamate in the nerve terminals above definite individual threshold can cause neurotoxicity and death of neurons via over activation of postsynaptic ionotropic glutamate receptors^30,31^. Also, data on changed neurotransmitter transport in the presence of MPs agreed with our recent studies regarding neurotoxic effects of water-suspended plastic smoke preparations^17,18^, where smoke preparations from polyethylene bottles showed almost similar changes (as compared to MPs) in L-[^14^C]glutamate and [^3^H]GABA transport in nerve terminals.

ROS are involved in the development of many human diseases and also perform physiological functions. ROS are counterbalanced by an antioxidant defense system that modulates ROS levels thereby allowing their physiological roles and minimizing the oxidative damage, which in turn provokes disease development^33^. It was suggested that the critical role in the redox balance maintenance belongs to antioxidant enzymes, but not small-molecule antioxidant compounds^34^. Here, it was shown that MPs decreased H_2_O_2_- and kainate-induced ROS generation (Fig. 9). These effects did not contradict our recent results obtained using plastic (polyethylene bottle) smoke preparations^17,18^ and data of literature, where antioxidant properties, in particular the ability to act as ROS scavenger were shown for carbon dots and plastic organics^35–39^. ROS are very important for the regulation of neurotransmission (especially GABA-ergic one) and synaptic plasticity^40^, an inhibition of kainate-induced ROS production by MPs may result in the disruption of receptor-mediated signaling pathways, thereby indirectly influencing neuronal activity. It can be speculated that MP-induced changes in ROS production may contribute to the alterations in the extracellular levels of [^3^H]GABA (and to less extent L-[^14^C] glutamate) in nerve terminal preparations (Table 2).

MP-induced changes in the membrane potential of nerve terminals and isolated neurons (Fig. 6) were in line with our previous experiments on wood smoke-induced changes in the membrane potential of nerve terminals and cell viability in culture (gut COLO 205 cells)^17,18,41^.

The question rose from the obtained results regarding a mode of neuroactive action of MPs in nerve terminals. Our recent study concerning membrane active capability of modelling carbon dots synthesized from β-alanine demonstrated that they increased transmembrane current of cations in the suspended bilayer lipid membrane (BLM) by inducing stable potential-dependent cation-selective pores^42^. This in turn was coincided with impaired synaptic neurotransmission, in particular the increased extracellular level of excitatory neurotransmitters L-[^14^C] glutamate, D-[^2,33^H] aspartate, and the inhibitory ones [^3^H] GABA, [^3^H] glycine in the nerve terminals. Carbon-containing nanoparticles synthesised by combustion of organics significantly dysregulated presynaptic processes^43,44^.

Therefore, it can be expected that carbon nanoparticle component of MPs directly (moving through the plasma membrane to the cytosol of nerve terminals) or/and indirectly (through an interaction with the plasma membrane of nerve terminals, disturbance of its integrity and membrane-associated pathways) can influence functioning of intracellular organelles, such as mitochondria and synaptic vesicles. Indeed, the experiments using JC-1, a fluorescent cationic carbocyanine dye accumulated by mitochondria, showed that the mitochondrial potential was significantly (appx twice) dissipated by MPs (Fig. 7). Also, the measurements of synaptic vesicle acidification using a pH-sensitive fluorescent probe acridine orange revealed a dissipation of the proton gradient of synaptic vesicles in response to the application of MPs (Fig. 8). The principal difference between these two intracellular organelles regarding their potential interaction with MP-derived carbon nanoparticle component was the following: MP-induced pores formed by the nanoparticle component in the plasma membrane of the nerve terminals can be transferred to synaptic vesicles during permanent synaptic vesicle recycling, in contrast to mitochondria, where the nanoparticles should penetrate the plasma membrane to reach mitochondria and interact with the mitochondrial membrane. Also, it is not excluded that besides carbon nanoparticle components, other smoke compounds can also contribute to mitochondria response. In perspective, our further efforts will be focused on analysis of changes in mitochondria functioning in response to application of smoke preparations from different types of organics. Also, further experiments using in vivo animal models, inhalation approaches and new technique^45–47^ are necessary to uncover detailed mechanisms of MPs action.

It is clear from our results that the neurotoxic effects of MPs were almost similar to the main neuroactive characteristics of smoke preparations synthesized from polyethylene bottles^17,18^. Therefore, accidental or intentional utilization of medical facemask waste by combustion can contribute to plastic-related air pollution of the environment. It has been suggested that the central nervous system is not substantially protected against evolutionary new challenges such as exposure to anthropogenic carbon-containing air pollution PM^32^, moreover an association of air pollution by PM and expansion of neurological/neurodegenerative disorders/disease was already proven^4,48^. Particles, especially nano-sized ones, can be deposited in humans in the nasal region, and move along the olfactory nerve axons straight to the brain, thereby circumstancing the blood brain barrier^49^. Our data can be useful in complex environment monitoring as potential essential variables for air quality estimation^50^.

Besides air pollution, PM released during facemask combustion can also contribute to plastic-related pollution of water resources^51^. MPs contained nano- and micro-particles (Fig. 1), and in situ these particles in combination with incompletely combusted ones can be suspended in water that occurs during rains, floods, fire control and extinguishing. Carbon-containing particles can become water soluble, and contaminate water resources, sea and ocean^52^. The plastic-derived particles can interact with fish and murine organisms, and then after feeding they can accumulate in humans causing health threats. Despite utilization problems, plastic is currently used to produce many products because of its low cost, light weight, and high durability and is emerging as the dominant material causing marine pollution^51,53–55^. It should be noted that PM can be neurotoxic itself and also due to adsorption of neurotoxic environmental components, in particular heavy metals^45,47^.

## Methods and materials

### Materials

EGTA, EDTA, HEPES, Ficoll 400, aminooxiacetic acid, d-glucose, sucrose, Whatman GF/C filters, the fluorescent dye 2′,7′-dichlorofluorescein, Sigma-Fluor® High Performance LSC Cocktail, organic counting scintillant (OCS) and the analytical grade salts were purchased from Sigma (St. Louis, MO, USA); L-[^14^C(U)] glutamate, [^3^H]GABA (γ-[2,3-^3^H(N)]-aminobutyric acid) were from Perkin Elmer (Waltham, MA, USA). Rhodamine 6G and acridine orange were obtained from Molecular Probes (USA). JC-1, Mitochondrial Membrane Potential Assay Kit was from Novusbio.

### Collection of smoke aerosol from combustion of disposable medical facemask under laboratory conditions

Disposable medical facemasks used in this study were composed of polypropylene fibers^16^. Smoke from combustion of the facemasks was collected in the laboratory conditions during the entire flaming, mixed combustion phase, when the flaming and smoldering phases were present simultaneously, and smoldering phase. In particular, the facemasks (60 g) were combusted; smoke emissions were collected using a specially designed vacuum installation and bubbled through 48 ml of water. Water-suspended MPs enriched with the most neuroactive nano-sized PM^32^ were obtained by filtration through glass microfiber filters with a pore diameter of 1.0 µm^17^. Obtained MPs were dried to measure the concentration of particulate smoke components. A concentration of dry matter in MPs was 700 µg/ml.

### Physical, optical, fluorescent and surface properties of MPs

#### Dynamic light scattering

Particle size in MPs was examined using dynamic light scattering with a laser correlation spectrometer Zetasizer Nano ZS, Malvern Instruments (UK) equipped with He–Ne laser (P_max_ = 4 mW, 633 nm).

#### Zeta potential

Z-potential of MP nanoparticles was determined using laser Doppler electrophoresis technique that was inbuilt in the Zetasizer Nano ZS, Malvern Instruments (UK). Measuring procedures were carried out in capillary cell DTS1070 at such parameters: scattered angle 17°; refractive index (RI) = 1.450, like as for carbon nanoparticles; dispersant – water (T = 25 °C, viscosity 0.8872 cP, RI = 1.330); Henry’s function (ƒ(K_α_)) = 1.5 (took according Smoluchowski approximation for aqueous solutions); number of measurements − 5 (each measurement includes 50 runs for 10 s).

#### Optical and fluorescent properties

Absorption characteristics of MP colloid were investigated using UV–Vis spectrophotometer Cary Eclipse Varian 50 Scan with wavelength range limits from 190 to 1100 nm. Measurements were conducted in Hellma Analytics Quartz SUPRASIL® High Precision Cell (10 × 10 mm light path).

Fluorescent properties of samples were determined using Agilent**®** Cary Eclipse spectrofluorimeter, equipped with a 75 kW Xenon flash lamp with Δpulse = 2 μs, two Czerny-Turner type monochromators and an 800 V PM detector. The lamp frequency is set to 100 Hz by default.

### Raman and FTIR spectroscopy

The Raman spectra of MPs were monitored using a custom-made Raman instrument pre-verified, and equipped with a 40 × objective microscope. A Verdi G laser, Coherent Inc. with a wavelength of 532 nm was applied to excite MPs, the laser power on the sample was 30 mW, and the scattered light of samples was collected by the lens and filtered using two filters (RazorEdge 0° Longpass filter, Semrock). Raman scattered light was focused on a monochromator inlet slit (IsoPlane 320, Princeton Instruments) set at 30 μm to deflect unfocused light, and have a high spectral resolution. The monochromator was equipped with a diffraction grating of 600 lines/mm, and calculated spectral resolution was appx 2 cm^−1^. A CCD (PyLoN: 400BR-eXcelon CCD, Princeton Instruments), cryogenically cooled at − 120 °C was used as a detector^18–26^.

MPs were analysed using FTIR spectrometer INVENIO-R (Bruker, Germany). The samples for the Raman scattering spectra, and infrared absorption were made by applying a drop of MP solution to a CaF_2_ substrate^18^.

### Neurochemical study of MPs

#### Ethical statement in animal experiments

Wistar rats, males, body weight of appx 100–120 g, were kept in a temperature-controlled room at 22–23 °C using the institute`s animal facilities, where the rats were provided ad libitum with water and dry food. Experiments involving animals were conducted in accordance with the Guidelines of the European Community (2010/63/EU), the ARRIVE guidelines for reporting experiments involving animals^56,57^ and local laws/policies. The protocol was approved by the Animal Care and Use Committee of the Palladin Institute of Biochemistry, National Academy of Sciences of Ukraine, the Protocol #1 from 28/01/2021. To follow a reduced animal number strategy, synaptosomal preparations were shared between radiolabeled and fluorescence experiments. The total number of rats used in this study was 15, among them 15 rats were used for L-[^14^C]glutamate and [^3^H]GABA transportation analysis; 15 shared rats − fluorescence measurements.

#### Isolation of the rat cortex nerve terminals (synaptosomes)

The synaptosomal preparations were obtained using differential and Ficoll-400 density gradient centrifugation of rat brain homogenates according to the method described by Cotman with minor modifications^58^. The protein concentrations were recorded according to Larson^59^. MP aliquots were added to the synaptosomal suspensions and incubated for 10 min.

#### Isolation of neurons from the rat cortex

The rat cortex was dissected and connective tissue was removed. One cortex was used to obtain one preparation of neurons. The tissue was incubated in HEPES-buffered saline (10 mM HEPES, 150 mM NaCl, pH 7.4), then tissue was transferred to polystyrene tubes and incubated with protease from *Streptomyces griseus* type XIV using a shaking water bath at 30 °C. After inactivation of protease and washing procedure, the neurons were re-suspended using the siliconized 9-in Pasteur pipette^60^.

#### The transporter-mediated uptake of L-[^14^C]glutamate by the nerve terminals

The initial rate of L-[^14^C]glutamate uptake and its accumulation by synaptosomes was measured as follows. Synaptosome suspensions (125 μl of suspension, 0.4 mg of protein/ml) were pre-incubated with MP aliquots at 37 °C for 10 min. The uptake was initiated by the application of L-glutamate and L-[^14^C]glutamate (10 µM and 450 nM, 0.167 µCi/ml, respectively). The synaptosomes were further incubated at 37 °C for 1 min (the initial rate) and 10 min (the accumulation), and then sedimented using a microcentrifuge (20 s at 10,000×*g*). Nonspecific binding of L-[^14^C]glutamate was assessed in cooled samples after addition of radioactivity. L-[^14^C]glutamate uptake was assessed as a decrease in radioactivity in the aliquots of supernatants (100 μl) and an increase in radioactivity in SDS-treated pellets^61^. L-[^14^C]glutamate uptake was calculated with Sigma-Fluor® High Performance LSC Cocktail (1.5 ml) using liquid scintillation counter Hidex 600SL (Finland). L-[^14^C]glutamate uptake data were collected in triplicate from several (n) experiments performed with different synaptosome preparations.

#### The transporter-mediated uptake of [^3^H]GABA by the nerve terminals

The synaptosomes were diluted by the standard salt solution containing aminooxyacetic acid (100 μM), the protein concentration was 200 μg/ml. The synaptosome preparations were pre-incubated at 37 °C for 10 min, MP aliquots were added to the synaptosome suspensions, and the samples were further incubated for 10 min. The uptake was initiated by the application of GABA and [^3^H]GABA (1 μM or 50 nM, 4.7 μCi/ml, respectively). To determine the initial rate of [^3^H]GABA uptake, the process was completed in 1 min by filtering aliquots through Whatman GF/C filters. After washing, the filters were dried, and then were suspended in the organic counting scintillant and counted using liquid scintillation counter Hidex 600SL (Finland). Nonspecific binding of [^3^H]GABA was evaluated in cooled samples filtered immediately after the addition of radioactivity^62^. [^3^H]GABA uptake data were collected in triplicate from several (n) experiments performed with different synaptosome preparations.

#### The exocytotic release and the extracellular level of L-[^14^C]glutamate in the nerve terminal preparations

The synaptosome preparations (2 mg of protein/ml) after pre-incubation at 37 °C for 10 min were loaded with L-[^14^C]glutamate (2.81 μM, 1 μCi/ml) at 37 °C for 10 min. After loading procedure**,** the synaptosome suspensions were washed with 10 volumes of ice-cold standard salt solution; the pellets were re-suspended to reach a final concentration of 1 mg of protein/ml. Synaptosome suspensions (125 μl; 0.5 mg of protein/ml) were pre-incubated at 37 °C for 10 min, then MP aliquots were added and incubated with synaptosomes for 10 min, and then sedimented using a microcentrifuge (20 s at 10,000 g). The extracellular level of L-[^14^C]glutamate in the nerve terminal preparations was measured at 6 min time point. The exocytotic release of L-[^14^C]glutamate stimulated by membrane depolarization with 35 mM KCl, was measured at 6 min time point and calculated by subtraction of the release values in Ca^2+^-containing medium and Ca^2+^-free one. The extracellular level of L-[^14^C]glutamate and its release were recorded in the aliquots of supernatants (100 μl) and pellets using liquid scintillation counter Hidex 600SL (Finland) and Sigma-Fluor® High Performance LSC Cocktail (1.5 ml), and the values were expressed as the percentage of total accumulated synaptosome label^63^. L-[^14^C]glutamate release data were collected in triplicate from several (n) experiments performed with different synaptosome preparations.

#### The exocytotic and tonic release of [^3^H]GABA and the extracellular level of [^3^H]GABA in the nerve terminal preparations

The synaptosome preparations (2 mg of protein/ml) were pre-incubated at 37 °C for 10 min and loaded with [^3^H]GABA (50 nM, 4.7 μCi/ml) for 10 min. GABA transaminase inhibitor aminooxyacetic acid (100 μM) was used during [^3^H]GABA loading and release experiments to minimize the formation of GABA metabolites. After loading, the synaptosome suspensions were washed with 10 volumes of ice-cold standard salt solution, the pellets were re-suspended to have protein concentration of 1 mg of protein/ml. Synaptosome suspensions (120 μl) were pre-incubated at 37 °C for 10 min, then the aliquots of MPs were added and incubated for 10 min, and sedimented using a microcentrifuge (20 s at 10,000 g). The extracellular level of [^3^H]GABA in synaptosome preparations was recorded at 6 min time point. Tonic leakage of [^3^H]GABA from the nerve terminals was recorded during simultaneous blockage of GABA transporters by NO-711 (30 μM), and expressed as NO-711-induced increase in the extracellular level of [^3^H] GABA. The exocytotic release of [^3^H]GABA was stimulated by 15 mM KCl and measured in the presence of NO-711 (30 μM) in Ca^2+^-containing medium at 6 min time point. [^3^H]GABA radioactivity was measured in the aliquots of supernatants (90 µl) using liquid scintillation counter Hidex 600SL (Finland) and Sigma-Fluor® High Performance LSC Cocktail (1.5 ml), and the values were expressed as the percentage of total accumulated synaptosome [^3^H]GABA^63^. [^3^H]GABA release data were collected in triplicate from several (n) experiments performed with different synaptosome preparations.

#### The membrane potential of the nerve terminals and neurons

The membrane potential of the nerve terminals and neurons was monitored using the fluorescent potentiometric dye rhodamine 6G (0.5 µM) based on its potential-mediated binding to the membranes^63^ The synaptosome or neuron suspensions (0.2 mg of protein/ml) were pre-incubated at 37 °C for 10 min, and the measurements were carried out using a thermostated cuvette with continuous stirring. The synaptosome preparations and suspensions of neurons were equilibrated with the probe, and after that MP aliquots were added. The ratio (*F*) as an index of the membrane potential was calculated according to equation *F* = *F*_t_/*F*_0,_ where *F*_0_ and *F*_*t*_ were the fluorescence intensities of rhodamine 6G in the absence and presence of the synaptosomes and neurons, respectively. *F*_0_ was estimated by the extrapolation of the exponential decay function to *t* = 0. The fluorescence measurements were carried out using a fluorescence spectrofluorometer QuantaMaster™ 40 (PTI, Inc., Canada) and a Hitachi 650-10S spectrofluorimeter at 528 nm (excitation) and 551 nm (emission) wavelengths.

#### The mitochondrial membrane potential of the nerve terminals

The cationic membrane-permeable dye JC-1, Mitochondrial Membrane Potential Assay Kit, was used to evaluate changes in the mitochondrial membrane potential of the nerve terminals. JC-1 showed potential-dependent accumulation by mitochondria, as evidenced by green fluorescence emission at (~ 529 nm) for the monomeric form of the probe, which was red-shifted (~ 590 nm) with concentration-dependent formation of red fluorescence J-aggregates^64^. The suspension of synaptosomes was incubated in a cuvette with a magnetic stirrer, the final protein concentration of which was 0.15 mg/ml, then JC-1 was added up to a final concentration of 2 µM, incubated in the dark at 37 °C for 10 min, and then the spectrum of the probe fluorescence was recorded at an excitation wavelength of 485 nm and an emission wavelength from 510 to 610 nm. Then, MP aliquots were added to the cuvette and the kinetics of probe fluorescence was monitored for 30 min at an excitation wavelength of 485 nm and an emission wavelength of 590 nm. Then, the MP-changed spectrum of the probe fluorescence was recorded at an excitation wavelength of 485 nm and an emission wavelength from 510 to 610 nm. For quantitative analysis, the ratio of the fluorescence intensity at 590 versus 530 nm was used, which allows to ignore the difference in the probe loading, the number of mitochondria and the probe fluorescence quenching by MPs. The fluorescence measurements were performed on a Hitachi 650-10S spectrofluorimeter.

#### Synaptic vesicle acidification in the nerve terminals

A pH-sensitive fluorescent dye, acridine orange, was selectively accumulated by the acid compartments of the nerve terminals, i.e., synaptic vesicles^65^. The changes in the dye fluorescence were measured using spectrofluorometer PTI QuantaMaster40 and Hitachi 650-10S at excitation and emission wavelengths of 490 and 530 nm, respectively. The reaction was started by the application of acridine orange (a final concentration of 5 μM) to synaptosome suspensions (a final concentration of 0.2 mg of protein/ml) pre-incubated in a stirred thermostatted cuvette at 37 °C for 10 min. Fluorescence (F) was calculated according to: *F* = *F*_t_/*F*_0_, where *F*_0_ and *F*_*t*_ were fluorescence intensities of the dye in the absence and presence of the nerve terminals, respectively. *F*_0_ was calculated by extrapolation of exponential decay function to *t* = 0.

#### ROS in the nerve terminals

A cell-permeable non-fluorescent probe, 2′,7′-dichlorodihydro-fluoresceindiacetate (H2-DCFDA) was applied to monitor ROS production in the nerve terminals^17,18^. The probe became highly fluorescent upon oxidation after the intracellular de-esterification. The synaptosome suspensions (a final concentration of 0.2 mg of protein/ml) were incubated with H2-DCFDA (a final concentration of 5 μM) for 5 min in a stirred thermostated cuvette at 37 °C, and then the kinetic measurements were carried out. 2′,7′-dichlorofluorescein (DCF) fluorescence changes were documented at excitation and emission wavelengths of 502 and 525 nm, respectively, (slit bands were 2 nm each) using a fluorescence spectrofluorometer QuantaMaster™ 40 (PTI, Inc., Canada).

#### Inclusion and exclusion criteria

The “n” value is present in the Figures and Tables for each experimental group of rats, and there were no exclusions among the experimental groups. The quality of synaptosomal isolation procedure from rat brains and synaptosome viability were controlled using specific criteria, i.e. the extracellular synaptosomal level of L-[^14^C]glutamate, that characterized dynamic glutamate turnover across the plasma membrane of the nerve terminals^30,31^. No data points were excluded from analyses because of their biologically implausible values.

#### Statistical analysis

The results were expressed as the mean ± S.E.M. of *n* independent experiments. Experimental data on the effects of control vs. MPs were analysed using one-way ANOVA. The accepted level of significance was set at *p* < 0.05.

## Conclusions

In summary, taking into account the importance of the problem associated with utilization of disposable medical facemask waste deposits^11^, the optical, surface and neuroactive properties of smoke PM released to the environment during the combustion of the facemasks were analyzed. Studying “cross-check” neurotransmission parameters at the presynaptic site, it was revealed that MPs significantly affected key characteristics of glutamate- and GABAergic synaptic neurotransmissions, and also decreased the membrane potential of the nerve terminals and neurons, mitochondrial potential, synaptic vesicle acidification, and hydrogen peroxide- and kainate-induced ROS generation in the nerve terminals. Neurotoxic signs of MPs in the nerve terminals very resembled effects of plastic (polyethylene bottle) smoke preparations shown in our previous study^17,18^. Therefore, accidental or intentional utilization of the facemask deposits by combustion can contribute to neurological plastic-related health hazards through environmental pollution of air and water resources.

## Data Availability

The datasets used during the current study are available from the corresponding author upon reasonable request.

## Abbreviations

PM: Particulate matter
DCF: 2′,7′-Dichlorofluorescein
EDTA: Ethylenediaminetetraacetic acid
GABA: γ-Aminobutryic acid
HEPES: 4-(2-Hydroxyethyl)-1-piperazineethanesulfonic acid
MP: Disposable medical facemask smoke aerosol preparations
ROS: Reactive oxygen species
UV–Vis: Ultraviolet–visible
